# Bromoform in the Treatment of Whoopingcough

**Published:** 1893-05

**Authors:** 


BROMOFORM IN THE TREATMENT OF WHOOPINGCOUGH.—In the February number of the *Practitioner* Mr. F. W. Burton-Fanning, of the Jenny Lind Infirmary, Norwich, reports his experience in the treatment of thirty cases of whoopingcough with bromoform. Except for one death—that of an infant whose whoopingcough was complicated with capillary bronchitis, whose condition was desperate when the treatment was begun, and who retained only one dose of the medicine—the results are said to have been uniformly gratifying. The dosage of bromoform recommended is as follows: For children under a year old, half a minim, three times a day; for those from a year to three years old, a minim; and for those from three to six years old, two minims. If necessary, these doses may safely be increased gradually until they are doubled. The mixture used by Dr. Burton-Fanning consisted of a minim of bromoform, half a drachm of compound powder of tragacanth, half a fluidrachm of syrup, and water enough to make half a fluidounce. Bromoform, which should be colorless, becomes brown on exposure to light; then it should on no account be used. The mixture mentioned should be thoroughly shaken before a dose is given.—*Ex.*

